# Implicit affectivity in clinically depressed patients during acute illness and recovery

**DOI:** 10.1186/s12888-019-2365-3

**Published:** 2019-11-29

**Authors:** Thomas Suslow, Charlott Maria Bodenschatz, Anette Kersting, Markus Quirin, Vivien Günther

**Affiliations:** 1 Department of Psychosomatic Medicine and Psychotherapy, University of Leipzig, Semmelweisstr. 10, 04103 Leipzig, Germany; 2 LIFE Leipzig Research Centre for Civilisation Diseases, University of Leipzig, 04103 Leipzig, Germany; 3 Department of Psychology, Technical University München, 80333 München, Germany; 4 PFH Göttingen, 37073 Göttingen, Germany

**Keywords:** Depression, Implicit affect, Explicit affect, Course of illness, Longitudinal study, Implicit Positive and Negative Affect Test

## Abstract

**Background:**

Clinical depression is characterized by high levels of negative affect (NA) and attenuated positive affect (PA). Psychological and pharmacological treatments have been shown to reduce NA and to enhance PA in depressed patients. Following dual-process models, two types of affect can be distinguished: explicit (or self-reported) affect, which is formed by conscious reflections, and implicit affect, which relates to automatic affective reactions. The present study was conducted to examine, for the first time, both implicit and explicit affectivity in patients suffering from acute depression. Moreover, changes in patients’ implicit and explicit affectivity were investigated over the course of inpatient treatment.

**Methods:**

Thirty-nine patients suffering from major depression and 39 healthy individuals participated in the study. Implicit affectivity was assessed using the Implicit Positive and Negative Affect Test. The explicit state and trait affectivity were measured by the Positive and Negative Affect Schedule. The level of depressive symptoms was assessed with the Beck Depression Inventory. Tests were administered to patients after admission and after 7 weeks of therapy, whereas healthy controls were investigated only once. We examined whether either comorbidity or antidepressant medication has an effect on affectivity.

**Results:**

Patients with acute depression had lower implicit and explicit PA scores and higher implicit and explicit NA scores than the healthy controls. After treatment, patients’ level of depression decreased significantly. At posttreatment, patients exhibited heightened implicit and explicit PA and diminished explicit trait NA. Independent of antidepressant medication and comorbidity, no significant change in implicit NA was observed over the course of treatment. Implicit NA was correlated with explicit NA in acute depression but not during recovery.

**Conclusions:**

Acute depression appears to be characterized by decreased implicit and explicit PA and increased implicit and explicit NA. After 7 weeks of treatment, depressed patients’ implicit and explicit PA increased, and explicit trait NA decreased. No decrease in implicit NA and explicit state NA occurred over the course of treatment. Finally, it seems that in the state of acute depression, the interplay between the automatic and reflective systems could be increased for negative affectivity.

## Background

The essential features of major depressive disorder are a prolonged depressed mood and diminished interest or pleasure in response to situations or stimuli that previously generated positive emotions. Watson and Tellegen [1] proposed that current and habitual emotional experiences can be described as two independent dimensions: positive and negative affect. Negative affect (NA) refers to emotional distress and a broad range of negative emotional states, such as sadness, guilt, and fear, whereas positive affect (PA) describes the extent to which a person feels happy, interested, and active. In healthy individuals, there is a clear preponderance of PA in everyday life: PA is experienced far more frequently and intensely than NA [2, 3]. Depression is characterized by high levels of NA and the co-occurrence of attenuated PA [4, 5]. These associations between depressive symptoms with low PA and high NA have been documented in numerous studies [6–8]. When asked on a daily basis, depressed patients reported, on average, less positive and heightened negative emotions in their natural environment relative to the healthy controls [9, 10].

There is growing evidence that the impaired ratio between PA and NA in depression can be altered by psychological and pharmacological treatments. Oren-Yagoda et al. [11] demonstrated increases in PA and decreases in NA over the course of a cognitive behavior therapy combined with pharmacological treatment, whereas Kring et al. [7] only found improvement in NA and depressive symptoms but not in PA. Using a daily monitoring procedure in patients’ natural living environment, Eddington et al. [12] showed significant changes in positive and negative mood after different forms of psychotherapy, with PA demonstrating even larger effects than NA. Enhanced PA [13] and declined NA in depression have also been reported after treatment with antidepressant medication [14].

According to dual-process models, two types of information processing systems can be assumed: a reflective and conscious fully accessible system, which processes information sequentially and an impulsive, and a not directly conscious accessible system, which processes information automatically and in parallel [15]. The neural mechanisms of these systems have also been documented (for the case of affect, see [16, 17]). Self-report tests of affectivity assess conscious affective experiences that have been termed *explicit* affect. Explicit affect is formed by conscious reflections and comparisons between affective episodes [18], also implicating the effects of feeling and emotional display norms. Conversely, implicit affect relates to processes of the impulsive system encompassing spontaneous affective reactions. These processes are thought to involve the activation of a large amount of affective information simultaneously, such as episodic and declarative memories [18].

However, individuals can become aware of aspects of intuitive affective processes through information exchange with the reflective processing system. Thus, to some degree, there exists an interplay between systems. According to Quirin and Lane [19], implicit affect can influence and be integrated into explicit affective experience. The authors suppose that visceromotor and somatomotor manifestations of emotion occur frequently in the absence of conscious emotional experience. However, implicit affect appears to constitute an important foundation on which more differentiated and conscious emotional experience is built. Quirin et al. [18] showed that when the spontaneity of response is requested during the completion of an explicit affect scale (i.e., individuals’ judgments are spontaneously guided by gut feelings) the association of explicit affect with implicit affect is substantially stronger than under conditions of reflective responding. The ventromedial prefrontal cortex (PFC) in concert with the amygdala, thalamus and insula might be crucially involved in the generation of implicit affect, whereas the dorsal anterior cingulate gyrus and the dorsomedial PFC seem to play important roles in the conscious processing and reflection of affective states [19, 20].

Implicit affect can be measured using indirect assessment methods such as the Implicit Positive and Negative Affect Test (IPANAT [18, 20]). In the IPANAT, participants assess the extent to which nonsense words, which purportedly originate from an artificial language, express positive and negative feelings. The IPANAT consists of two scales, measuring implicit *positive* affect and implicit *negative* affect, that are largely independent of each other [18]. The IPANAT is a reliable instrument capturing the trait and state aspects of implicit affectivity [20]. There is evidence for the convergent and discriminant validity of the IPANAT and its two-dimensional model of implicit positive versus negative affect [18]. Since its introduction, the IPANAT has been translated into more than 10 languages and has been widely distributed in recent years [21]. Thus, both scores provided by the IPANAT, implicit positive affect and implicit negative affect, comprise trait and state variance. To summarize, the dimensions of positive and negative affect underlie explicit and implicit affective experiences. Implicit positive affect has been found to correlate with explicit positive state and trait affect, and implicit negative affect has been observed to correlate with explicit negative state and trait affect in healthy individuals [18]. The investigation of implicit affect does not replace the study of explicit affect but, rather, is thought to complement and expand it.

The IPANAT has been shown to be a valuable predictor of spontaneous behavioral and psychophysiological reactions to emotion stimuli and stressors in healthy individuals. Implicit NA, as assessed by the IPANAT, is associated with unintentionally occurring processes of attention allocation to dysphoric stimuli [22]. These findings demonstrate the utility of the IPANAT in investigating individual differences in depression-relevant attentional biases and cognitive vulnerability. Moreover, the IPANAT’s NA was found to be related to the detection of and neural responses to threatening stimuli in the brain regions involved in fear and flight behavior [23]. High implicit negative affectivity predicted cortisol response to acute stressors, whereas low implicit PA predicted circadian cortisol release [24]. Similarly, another study reported that implicit PA was negatively associated with cortisol levels in daily life [25]. Furthermore, implicit affectivity was found to be associated with recovery from stress-contingent blood pressure increases [26, 27]. In the abovementioned studies, the IPANAT was found to predict spontaneous behavioral and psychophysiological reactions to stress or emotion stimuli exclusively or above and beyond the explicit measures of affect, underscoring its usefulness in emotion research.

The aim of the present study was to examine, for the first time, both implicit and explicit affectivity in patients suffering from acute clinical depression. We hypothesized that depressed patients differ from healthy control subjects concerning positive and negative affect. Specifically, on the basis of previous findings on explicit affectivity, it was expected that depressed patients would manifest lower positive state and trait affect scores and higher negative state and trait affect scores than healthy controls. Similarly, it was hypothesized that depressed patients exhibit heightened negative implicit affect and reduced positive implicit affect compared to healthy individuals. A second aim of our study was to investigate changes in the implicit (and explicit) affectivity of depressed patients over the course of a naturalistic inpatient treatment program. Therefore, patients were reexamined after 7 weeks. We expected that depressed patients’ implicit and explicit positive affectivity increases, and that implicit and explicit negative affectivity decreases over the treatment program. Whether posttreatment patients show a prevalence of PA versus NA, as typically observed in healthy individuals, was explored. Finally, we examined the relationships between implicit and explicit affectivity in acute and partially remitted depressed patients compared to healthy individuals.

## Methods

### Participants

The patient group included 39 inpatients (26 women and 13 men) with an acute major depression episode according to the Structured Clinical Interview for DSM-IV Axis I Disorders (SCID-I [28]). The exclusion criteria were age of 46 years and older, neurological diseases, a history of bipolar or psychotic disorders, and substance abuse or addiction within the previous 6 months. Suicide attempts or serious suicidal intentions were general contraindications for study participation. Patients were consecutively recruited from a routine treatment program in the Department for Psychosomatic Medicine and Psychotherapy of the University of Leipzig. All patients underwent a psychodynamic-interactional-oriented psychotherapy program. The therapeutic setting included three group and two individual therapy sessions per week. The important aspects of the inpatient treatment include receiving insight into interpersonal conflicts, improvement of self-observation, dealing with criticism, and identification, verbalization and communication of emotions to therapists and other patients. The therapy program was conducted by a trained treatment team, consisting of physicians and clinical psychologists, supervised by a senior physician and a senior psychologist. Twenty-seven patients (69%) were taking antidepressant medication at the first test session, and two were additionally treated with benzodiazepines. In the second test session, 28 patients were treated with antidepressants, whereas no patient received benzodiazepines. Five types of antidepressants were given (selective serotonin reuptake inhibitors (SSRIs), serotonin and norepinephrine reuptake inhibitors (SNRIs), norepinephrine-dopamine reuptake inhibitors (NDRI), noradrenergic and specific serotonergic antidepressants (NaSSAs) and tricyclic antidepressants (TCAs)).

The healthy control group consisted of 39 volunteers (26 women and 13 men) without a history of psychiatric diseases. Diagnoses of current or past psychiatric disorders were determined by the Mini International Neuropsychiatric Interview (MINI, [29]). The MINI was only used to screen the healthy control subjects. Further exclusion criteria were neurological diseases and age of 46 years and older. Healthy participants were recruited via online advertisements and public notices. The notices to recruit healthy controls were posted in public places such as libraries, supermarkets, and student residencies. The demographic characteristics of healthy controls and depressed patients are presented in Table 1. There were no group differences in age, *t*(58.81)^1^ = 0.49; *p* > .05. However, the patients reported, on average, a lower education level compared to the controls (see Table 1).

**Table 1:** Demographic and questionnaire characteristics of healthy controls and depressed patients at baseline (means and SD (in brackets))

Variable	Control group (*N* = 39)	Depressed patients (*N* = 39)	*p*
Age	31.10 (4.39)	31.82 (8.04)	n.s.
Gender (f/m)	26/13	26/13	–
Level of School Education			< .05 ^A,^*
*N* 9th grade	1	2	
*N* 10th grade	6	17	
*N* 12th grade	32	20	
Illness duration of current episode (in months)	–	12.72 (13.74)	–
Number of episodes	–	3.00 (2.00)	–
Lifetime hospitalization (weeks)	–	5.21 (8.17)	–
BDI-II	3.82 (4.67)	31.26 (9.69)	< .001*
PANAS-PA trait	36.42 (5.29)	21.79 (6.87)	< .001*
PANAS-PA state	31.38 (5.90)	20.95 (6.82)	< .001*
PANAS-NA trait	14.29 (3.10)	27.15 (7.16)	< .001*
PANAS-NA state	12.08 (1.95)	21.79 (7.96)	< .001*
IPANAT-PA	2.41 (0.34)	2.04 (0.42)	< .001*
IPANAT-NA	1.72 (0.37)	1.90 (0.38)	< .05*

^A^ χ 2 (2) = 8.36, *p* = 0.015; * Significant differences between groups

*BDI-II* Beck Depression Inventory II; *PANAS-PA* Positive Affect Scale of the Positive and Negative Affect Schedule, trait and state version; *PANAS-NA* Negative Affect Scale of the Positive and Negative Affect Schedule, trait and state versions; *IPANAT-PA* Implicit Positive and Negative Affect Test, positive affect; *IPANAT-NA* Implicit Positive and Negative Affect Test, negative affect

### Procedure and psychometric measures

This study was approved by the local ethics committee of the Medical School of the University. After a detailed explanation of the study, written informed consent was obtained from all participants. All subjects received financial compensation after completion of the tasks. Healthy control subjects served as the reference group and completed the questionnaires only once. Depressed patients were initially tested approximately 2 weeks after admission to the clinic (baseline, *M* = 2.23 weeks, *SD* = 0.81 weeks) and, on average, after 7 weeks of therapy (posttreatment, *M* = 6.74 weeks; *SD* = 0.66 weeks). The SCID-I [29] was administered in the first test session at baseline. No patient terminated the treatment program during the testing period.

For all participants, the severity of self-reported depressive symptoms was assessed with the revised version of the Beck Depression Inventory (BDI-II [30]). The BDI-II consists of 21 items assessing the severity of the typical symptoms of depression, such as low mood, self-accusation, insomnia, and fatigue. Participants are asked to pick one of four statements of increasing intensity within the symptom domain. A rating of 0 indicates the absence of a symptom, whereas a rating of 3 indicates a severe symptom (for example, I do not feel sad (0), I feel sad much of the time (1), I am sad all the time (2), and I am so sad or unhappy that I can’t stand it (3)). The BDI-II exhibited good internal consistencies (α > 0.80). As an explicit measure of state and trait affect, the 20-item Positive and Negative Affect Schedule (PANAS [31]) was administered. Participants rated on a 5-point scale (1 = not at all, 5 = extremely) to what extent they feel different moods described by certain adjectives (e.g., interested, active, distressed, and nervous) in general (trait) and at the moment (state). The PANAS scales showed sufficient to good internal consistencies (α > 0.65). One healthy participant did not complete the trait version of the PANAS. Implicit affectivity was assessed by the Implicit Positive and Negative Affect Test (IPANAT [18]). The IPANAT is an indirect measure of affect, where participants are asked to rate the degree to which artificial words express certain positive and negative moods. Each of three positively and three negatively charged adjectives (helpless, tense, inhibited, happy, cheerful, and energetic) were presented along with each of the six words from a putative artificial language (e.g., VIKES and BELNI). Participants provided their judgments for the 36 word pairs on a 4-point scale (1 = doesn’t fit at all and 4 = fits well). The IPANAT has been shown to have satisfactory psychometric properties [18]. In the present patient and control samples, internal consistencies for the PA and NA subscales were *α* > .72, respectively. In healthy individuals, it has been shown that IPANAT scores remain relatively stable over different time periods when measured without preceding mood inductions [18]. There is also evidence that implicit affect, as measured by the IPANAT, could be altered by affect induction procedures. Hence, the IPANAT provides a suitable tool to assess both the trait and state components of positive and negative implicit affect (see [20] for an overview). Posttreatment data of the PANAS-PA trait and state and of the IPANAT are each missing for one patient.

## Results

### Group comparisons of explicit and implicit affectivity measures at baseline

The questionnaire characteristics of healthy controls and depressed patients at baseline are presented in Table 1. Independent *t*-tests revealed significant group differences in all affective measures. Compared to healthy controls, depressed patients demonstrated higher scores in depressive symptoms (BDI-II: *t*(54.75) = 15.92; *p* < .001), explicit NA (PANAS-NA trait: *t*(52.05) = 10.27; *p* < .001; state: *t*(42.55) = 7.40; *p* < .001), and implicit NA (IPANAT-NA: *t*(76) = 2.12; *p* < .05). Moreover, patients scored lower in explicit PA (PANAS-PA trait: *t*(75) = 10.45; *p* < .001; state: *t*(76) = 7.23; *p* < .001) and implicit PA (IPANAT-PA: *t*(76) = 4.34; *p* < .001).

Within the healthy group, individuals scored significantly higher in all PA scales when compared to the mean scores of the NA scales (PANAS trait: *t*(37) = 21.84; *p* < .001; PANAS state: *t*(38) = 19.40; *p* < .001; IPANAT: *t*(38) = 9.11; *p* < .001), indicating a prevalence of positive mood at explicit and implicit levels. In depressed patients, dependent *t*-tests revealed no significant differences between mean PA and NA scores (PANAS state: *t*(38) = 0.44; *p* > .05; IPANAT: *t*(38) = 1.42; *p* > .05), except for higher scores in explicit trait NA compared to PA (PANAS trait: *t*(38) = 2.71; *p* < .05).

### Comparisons of explicit and implicit affectivity measures between patients with and without medication

Unmedicated patients (*n* = 12) had higher implicit PA scores at baseline (2.25 (SD: 0.32) vs. 1.94 (SD: 0.43); *t*(37) = 2.25; *p* < .05) and reported more explicit positive state affect (25.92 (SD: 7.95) vs. 19.96 (SD: 5.68); *t*(37) = 2.69; *p* < .05)) than medicated patients (*n* = 27). No other group differences in explicit and implicit affectivity measures were revealed between medicated and unmedicated depressed patients.

### Comparisons of explicit and implicit affectivity measures between patients with and without comorbidity

Depressed patients with comorbid disorders (*n* = 13) did not differ from depressed patients without comorbid disorders (*n* = 26) in explicit and implicit affectivity measures with one exception. Patients with comorbidity had higher implicit PA scores at baseline than patients without comorbidity (2.14 (SD: 0.40) vs. 1.84 (SD: 0.39); *t*(37) = 2.21; *p* < .05).

### Change in explicit and implicit affectivity in depressed patients over time

Table 2 shows the mean scores and standard deviations for the affective scales at baseline and posttreatment in the depressed sample. Absolute changes in affective measures over time were tested using dependent *t*-tests. In the comparisons of pre- and posttreatment measures (concerning IPANAT NA, IPANAT PA, PANAS NA state, PANAS PA state, PANAS NA trait, PANAS PA trait, and BDI-II), we applied a Bonferroni correction for multiple comparisons to reduce the likelihood of type I error. Specifically, we divided the threshold of *p* < 0.05 by the number of critical tests (i.e., 7), resulting in a corrected threshold of *p* < 0.0071 (two-tailed). Depressive symptoms and explicit trait NA scores (but not explicit state NA) were significantly reduced at posttreatment (see Table 2). Moreover, significant increases in explicit positive affectivity could be observed. With respect to the IPANAT scales, a significant increase in implicit PA was revealed, whereas implicit NA did not significantly change over time (see Table 2).

**Table 2:** Questionnaire characteristics of depressed patients at baseline and posttreatment (means and SD (in brackets))

Variable	Depressed patients		*p*
	Baseline	Posttreatment	
BDI-II	31.26 (9.69)	18.41 (12.26)	< .001*
PANAS-PA trait	21.79 (6.87)	28.56 (8.15)	< .001*
PANAS-PA state	20.95 (6.82)	27.92 (9.66)	< .001*
PANAS-NA trait	27.15 (7.16)	21.93 (6.89)	< .001*
PANAS-NA state	21.79 (7.96)	18.56 (7.32)	n.s.
IPANAT-PA	2.04 (0.42)	2.36 (0.50)	< .001*
IPANAT-NA	1.90 (0.38)	1.78 (0.36)	n.s.

* significant at a threshold corrected for multiple comparisons of *p* < 0.0071 (two-tailed)

*BDI-II* Beck Depression Inventory II; *PANAS-PA* Positive Affect Scale of the Positive and Negative Affect Schedule, trait and state version; *PANAS-NA* Negative Affect Scale of the Positive and Negative Affect Schedule, trait and state versions; *IPANAT-PA* Implicit Positive and Negative Affect Test, positive affect; *IPANAT-NA* Implicit Positive and Negative Affect Test, negative affect

In the following explorative comparisons between patients with and without comorbidity and between medicated and unmedicated patients, a conventional statistical significance threshold of *p* < 0.05 was maintained. Patients with and without comorbidity both showed significant increases in implicit PA over time (2.14 (SD: 0.40) vs. 2.40 (SD: 0.42); *t*(24) = 2.73; *p* < .05 and 1.84 (SD: 0.39) vs. 2.28 (SD: 0.64); *t*(12) = 2.85; *p* < .05), but for both groups, no significant changes in implicit NA were observed. Moreover, both medicated and unmedicated depressed patients exhibited significant increases in implicit PA over time (1.94 (SD: 0.43) vs. 2.32 (SD: 0.56); *t*(26) = 3.45; *p* < .01 and 2.26 (SD: 0.33) vs. 2.44 (SD: 0.29); *t*(11) = 2.36; *p* < .05); for both groups, no significant changes in implicit NA were found.

At posttreatment, depressed patients had higher mean values in all explicit and implicit PA scales than in NA scales (PANAS state: *t*(37) = 3.76; *p* < .001; PANAS trait: *t*(37) = 3.07; *p* < .01; IPANAT: *t*(37) = 5.24; *p* < .001). These findings suggest a restored prevalence of positive mood, as could be observed in healthy controls.

### Correlations between implicit and explicit affectivity in healthy individuals

In the control subjects, implicit PA was correlated with explicit positive state and trait affect (*r* = .33, *p* < .05; *r* = .48, *p* < .01), but implicit NA was not significantly related to explicit negative state or trait affect.

### Correlations between implicit and explicit affectivity in depressed patients at baseline and posttreatment

At baseline, there were significant correlations of implicit PA with explicit positive state and trait affect (*r* = .37, *p* < .05; *r* = .47, *p* < .01) and of implicit NA with explicit negative state and trait affect (*r* = .53, *p* < .01; *r* = .34, *p* < .05) in the patient sample. At posttreatment, implicit PA was again correlated with explicit positive state and trait affect (*r* = .42 and .46, *p*s < .01), but implicit NA was not found to be correlated with explicit negative state or trait affect.

## Discussion

The main focus of the present study was on implicit affectivity in depressed patients during acute illness and recovery. In our study, the IPANAT [18] and self-report measures were administered two times, approximately 2 weeks after admission and after approximately 7 weeks of therapy, to assess implicit and explicit affectivity. Between baseline and posttreatment, all patients underwent psychodynamic-interactional-oriented psychotherapy, and more than two-thirds of the patients took antidepressants at both assessment points. After treatment, patients reported substantially fewer depressive symptoms. However, according to the BDI-II results, patients were on average still mildly depressed at posttreatment. The differentiation between implicit and explicit affect appears promising and theoretically valuable since they are assumed to refer to related but rather independent processing networks, an automatic system and a reflective system [15]. In a number of studies with healthy individuals, it was found that implicit affectivity, as measured by the IPANAT, predicts spontaneous behavioral and psychophysiological reactions to stress or emotion stimuli exclusively or above the explicit measures of affectivity [22–24, 26].

As expected, on an explicit level, acutely depressed patients reported more negative state and trait affects and less positive state and trait affects compared to healthy individuals. These findings are perfectly in line with those of many other questionnaire studies in the field [4, 5, 9, 10]. Importantly, our results suggest that acutely depressed patients also exhibit heightened implicit NA and reduced implicit PA in comparison with healthy subjects. Thus, according to our findings, acute depression appears to be characterized by decreased implicit and explicit PA and increased implicit and explicit NA. The pattern of affectivity on the implicit level seems to parallel that on the explicit level. The implicit affect results appear to indicate impaired automatic positive affective responsivity and heightened automatic negative affective responsivity in patients with acute depression. The data from neuroimaging research are consistent with this assumption, indicating automatic amygdala mood-congruent biases in terms of enhanced reactivity to negative emotional stimuli and reduced reactivity to positive emotional stimuli in clinical depression [32–34].

In our sample of healthy controls, a clear preponderance of PA in comparison to NA was observed in the self-report questionnaires as well as the IPANAT. This result is consistent with the notion that mental health is associated with more frequent and intense experiences of PA than of NA in everyday life [2, 3]. For patients suffering from acute depression, no prevalence of PA could be revealed on either the implicit or explicit level.

Our data confirm the hypotheses that after 7 weeks of inpatient treatment, depressed patients’ implicit and explicit PA increased, and their explicit trait NA decreased. Explicit state NA was not found to decrease over time. These results are consistent with the findings of intervention studies, demonstrating increases in PA and decreases in NA in depressed patients after psychotherapy or psychopharmacological treatment [11–14]. In this context, it is important to note that trait affects do not represent absolutely stable individual differences in the disposition to develop specific affective reactions. Trait affects are only of relative temporal stability, and changes occur in healthy individuals when, for example, challenging or burdensome responsibilities and new social roles are assumed [35]. Moreover, it has been shown that over the course of an antidepressant treatment, the dispositions to develop negative (i.e., neuroticism) and positive affects (as a facet of extraversion) change in depressed patients [36]. Thus, the remission or amelioration of depressive symptoms can diminish patients’ disposition to develop NA and increase the disposition to develop PA. Against this background, it is nevertheless somewhat surprising that our patients’ explicit state NA did not decrease from pretreatment to posttreatment. Possibly, the divergence of the findings concerning explicit state and explicit trait NA is due to the fact that the temporal reference period of the trait judgements is broader and, thus, may allow for a more reliable representation of a more general change or improvements in NA, whereas the state judgments register only the actual status at the time of testing. However, this point should not be overemphasized because conservative statistical thresholding led to nonsignificant results in the comparison of explicit state NA between pretreatment and posttreatment. At the conventional level of statistical significance (*p* < .05), a decrease in explicit state NA (but not in implicit NA) from pretreatment to posttreatment would have been revealed.

To our knowledge, our investigation is the first to examine the alterations of implicit affectivity in depression using an indirect psychometric test. However, no evidence was found in our study for a significant decrease in implicit NA over the course of treatment. It appears that NA may improve first on the explicit level and only later on the implicit level. Thus, changes in the automatically elicited negative affectivity of depressed patients may occur later and require more time to develop. Interestingly, this pattern of findings seems to not depend on antidepressant medication or comorbidity. Medicated and unmedicated patients as well as patients with and without comorbid disorders showed improvements in implicit PA but no changes in implicit NA over the course of treatment.

However, it has yet to be shown that implicit NA normalizes in the long term in depressed patients. It appears possible that, at least in some patients, heightened implicit NA may persist, which could be interpreted as vulnerability to developing NA. Implicit NA is thought to be linked to the automatic activation of the cognitive representation of negative affective experiences [20]. It has been observed that the number of previous episodes predicts the relapse and recurrence of depression. Depressive episodes seem to leave scars that increase vulnerability to new episodes [37]. Negatively biased cognitive processing in attention and memory seems to represent a stable vulnerability factor for depression [38]. Patients with remitted depressive disorder still show elevated emotional reactivity toward negative affective stimuli [39], even under nonconscious processing conditions [32]. Heightened implicit NA in remitted depressed patients might refer to their increased emotional responsivity to negative stimuli.

It has been recognized that deficiency in PA represents a core mechanism underlying depression [40]. The early improvement in PA rather than in NA predicted recovery from depression after pharmacotherapy [41]. Experience with PA seems to be a crucial factor facilitating remission from clinical depression [42, 43]. PA has beneficial effects, facilitating social contact and biasing attentional awareness toward positive cues in the environment [44]. Initial progress in PA may create a positive spiral of PA, which helps to finally reduce NA and depressive symptoms [45]. In healthy individuals, the boosting of implicit PA has been shown to be an important mechanism to deal with and recover from NA [46]. Future longitudinal studies with daily application of the IPANAT could help to obtain a detailed description of the implicit affect dynamics during recovery from depression and the precise role of implicit PA. Future prospective research might also further clarify the prognostic relevance of implicit NA concerning the course and outcome in depression. In a healthy sample, independent of the level of depressive symptoms, implicit NA was found to be a more relevant predictor of depression-related attentional biases than explicit NA [22].

The IPANAT, as an indirect measure of affectivity, could also represent a useful supplement to classic self-report measures in depression research, as it appears less amenable to distortions caused by reflections about one’s state, self-presentation or appellative tendencies [20]. Some patients might repress and minimize unwanted affect, while others may overreport negative affect [18].

Future research should investigate the alterations of affective experience in acute depression and during recovery on the level of *specific, discrete* affect. It has been argued that a certain number of basic emotions, such as joy, fear, sadness, or anger, universally exist across all human cultures [47]. To assess explicit discrete affects, self-report scales such as the Differential Emotions Scale (DES [48]) could be administered. The DES is a standardized instrument that reliably divides the descriptions of affective experience into validated, discrete categories of affect. An IPANAT variant is available for the assessment of implicit discrete affect [20]. Specifically, it would be interesting to clarify whether implicit sadness, implicit fear, and implicit anger are equally heightened in patients with acute depression in comparison to healthy individuals and diminish to a similar extent over time.

According to our findings, patients exhibited a prevalence of PA versus NA at posttreatment on both an implicit and explicit level. It appears that although no significant changes were observed in implicit NA over the course of treatment, the ratio between PA and NA tended to normalize. At posttreatment, depressed individuals showed a preponderance of implicit and explicit PA, which is characteristic of healthy individuals. Finally, the relationship between implicit and explicit affectivity was examined in acutely ill and partially remitted depressed patients in comparison to healthy individuals. Interestingly, implicit PA was related to explicit positive state and trait affect in healthy controls as well as in depressed patients at both assessment points. Moreover, for healthy controls and depressed patients in recovery, implicit NA was not found to be correlated with explicit negative state or trait affect, but in the state of acute depression, patients manifested correlations between implicit NA and explicit negative state and trait affect. Thus, in the state of acute depression, the interplay between the automatic and reflective systems could be increased for negative affectivity. This observation may be interpreted within the context of neuroimaging findings, suggesting that in clinical depression, the functional balance between the amygdala and prefrontal structures is impaired. The amygdala is hyperresponsive to negative stimuli in depression and biases perception and higher-order cognition, while prefrontal executive control is decreased [49]. During the successful treatment of depression activity in prefrontal areas, subserving cognitive control and reflective functions, appears to enhance, whereas the bottom-up influence of the amygdala diminishes [50].

Some limitations of the present study should be acknowledged. This limitation of our study is that the healthy control group was tested only once. Moreover, our sample consisted mainly of female patients. Thus, it would be important to examine, in future studies, the course of implicit and explicit affect, as a function of gender, with a focus on male patients. Since personality disorders may influence the experience and reporting of affectivity, the presence of personality disorders should be explicitly assessed and controlled using standardized diagnostic interviews in future research on the subject.

It has been demonstrated that clinical depression is associated with a substantially increased risk for coronary heart disease and myocardial infarction [51]. The underlying mechanisms that link depression and heart disease have not been fully elucidated thus far, but it is assumed that the altered activation of stress pathways, including the sympathetic nervous system and the hypothalamic-pituitary-adrenal axis, plays an important role in this context [52]. It appears that measures of implicit affectivity, such as the IPANAT, could be valuable research instruments to enhance our understanding of how emotions and stress influence daily physiological activity and, in the long term, may cause heart disease in patients with depression.

## Conclusions

In the present study, implicit and explicit affectivity were investigated in patients suffering from acute clinical depression and during remission. According to our results, acute depression is characterized by decreased implicit and explicit PA as well as by increased implicit and explicit NA. At posttreatment, after 7 weeks of inpatient treatment, depressed patients’ implicit and explicit PA increased, and explicit trait NA decreased. However, no evidence was found for a decrease in implicit NA over the course of treatment. Changes in automatically elicited negative affectivity may occur later in depressed patients and need more time to develop compared to affectivity on the explicit processing level. It appears also possible that, at least in some patients, heightened implicit NA may persist as a vulnerability to developing NA. It appears that in the state of acute depression, the interplay between the automatic and reflective systems could be increased for negative affectivity.

## Data Availability

Data supporting our findings will be shared upon request.

## Abbreviations

BDI-II: Beck Depression Inventory II
DES: Differential Emotions Scale
IPANAT: Implicit Positive and Negative Affect Test
IPANAT-NA: Implicit Positive and Negative Affect Test, negative affect
IPANAT-PA: Implicit Positive and Negative Affect Test, positive affect
MINI: Mini International Neuropsychiatric Interview
NA: Negative affect
NaSSAs: Noradrenergic and specific serotonergic antidepressants
NDRI: Norepinephrine-dopamine reuptake inhibitors
PA: Positive affect
PANAS: Positive and Negative Affect Schedule
PANAS-NA: Negative Affect Scale of the Positive and Negative Affect Schedule
PANAS-PA: Positive Affect Scale of the Positive and Negative Affect Schedule
PFC: Prefrontal cortex
SCID-I: Structured Clinical Interview for DSM-IV Axis I Disorders
SNRIs: Serotonin and norepinephrine reuptake inhibitors
SSRIs: Selective serotonin reuptake inhibitors
TCAs: Tricyclic antidepressants

## Footnotes

1 Degrees of freedom were adjusted if group variances were nonhomogeneous (according to Levene’s test of variance homogeneity).
